# LRR domain of NLRX1 protein delivery by dNP2 inhibits T cell functions and alleviates autoimmune encephalomyelitis

**DOI:** 10.7150/thno.43441

**Published:** 2020-02-10

**Authors:** Ja-Hyun Koo, Do-Hyun Kim, Donghun Cha, Min-Jong Kang, Je-Min Choi

**Affiliations:** 1 Department of Life Science, College of Natural Sciences, Hanyang University, Seoul 04763, Republic of Korea; 2 Research Institute for Natural Sciences, Hanyang University, Seoul 04763, Republic of Korea; 3 Section of Pulmonary, Critical Care and Sleep Medicine, Department of Internal Medicine, Yale University School of Medicine, New Haven, CT 06520, USA; 4 Research Institute for Convergence of Basic Sciences, Hanyang University, Seoul 04763, Republic of Korea

**Keywords:** NOD-like receptor family member X1, T cell, BBB-penetrating peptide, dNP2, experimental autoimmune encephalomyelitis

## Abstract

Multiple sclerosis (MS) is a demyelinating inflammatory disease of the central nervous system (CNS), which is a chronic progressive disease and is caused by uncontrolled activation of myelin antigen specific T cells. It has high unmet medical needs due to the difficulty of efficient drug delivery into the CNS to control tissue inflammation. In this study, we demonstrate that a fusion protein of NOD-like receptor family member X1 (NLRX1) and blood brain barrier (BBB)-permeable peptide, dNP2 ameliorates experimental autoimmune encephalomyelitis (EAE).

**Methods**: We purified recombinant LRR or NBD regions of NLRX1 protein conjugated with dNP2. To examine intracellular delivery efficiency of the recombinant protein, we incubated the proteins with Jurkat T cells or murine splenic T cells and their delivery efficiency was analyzed by flow cytometry. To investigate the therapeutic efficacy in an EAE model, we injected the recombinant protein into mice with 3 different treatment schemes e.g., prevention, semi-therapeutic, and therapeutic. To analyze their functional roles in T cells, we treated MACS-sorted naïve CD4 T cells with the proteins during their activation and differentiation into Th1, Th17, and Treg cells.

**Results**: dNP2-LRR protein treatment showed significantly higher delivery efficiency than TAT-LRR or LRR alone in Jurkat T cells and mouse splenic T cells. In all three treatment schemes of EAE experiments, dNP2-LRR administration showed ameliorated tissue inflammation and disease severity with reduced number of infiltrating T cells producing inflammatory cytokines such as IFNγ. In addition, dNP2-LRR inhibited T cell activation, cytokine production, and Th1 differentiation.

**Conclusion**: These results suggest that dNP2-LRR is a novel agent, which regulates effector T cell functions and could be a promising molecule for the treatment of CNS autoimmune diseases such as multiple sclerosis.

## Introduction

Multiple sclerosis (MS) is an autoimmune disease caused by inflammation in the central nervous system due to recognition of proteins expressed on neurons as antigens 1-3. Of the various immune cells involved in disease progression including macrophages, microglia, and B cells, Th1 and Th17 cells in particular are known to be the key cells involved in MS pathogenesis 4-6. Currently, MS treatment focuses on relieving symptoms since no curative medicine has been developed that can directly control the function of the key cells involved in disease pathogenesis, such as effector T cells 1, 7.

Systemic treatment with anti-inflammatory drugs has been known to have limited efficacy in patients with central nervous system (CNS) inflammation due to the difficulty of these drugs in penetrating the blood-brain barrier (BBB). To overcome this limitation, various BBB-penetrating methods have been reported such as viral vector, non-viral nanoparticles, exosomes and enhancers 8-11. Previously, we reported a novel BBB-permeable peptide dNP2, that could deliver the cytoplasmic domain of CTLA-4 12 or VIVIT peptide 13 into T cells infiltrating into the CNS and thereby control autoimmune encephalomyelitis. Utilizing a BBB-penetrating peptide such as dNP2 would alter intracellular signaling events by appropriate cargo in immune cells, and hence control CNS-inflammatory diseases.

NLRX1 is a mitochondrial protein identified as a negative regulator of antiviral responses by regulating mitochondrial antiviral-signaling protein (MAVS)-interferon regulatory factor (IRF) 14, and stimulator of interferon genes (STING)-IRF signaling 15. It also plays a role in the regulation of autophagy by interacting with Tu translation elongation factor (TUFM) 16. In addition, it has also been reported as a negative regulator of TNF receptor associated factor 6 (TRAF6), and nuclear factor kappa-light-chain-enhancer of activated B cells (NF-κB) 17, 18. Recently, NLRX1 deficient mice have been studied in colitis 19 and EAE models 20, which are T cell-mediated diseases. NLRX1 deficient mice showed more severe colitis and EAE than WT mice with increased T cell infiltration and inflammatory cytokines. In another report, NLRX1 deficient 2D2 mice spontaneously developed EAE with an incident rate of over 50% 21. However, the potential role of NLRX1 as a therapeutic drug molecule to regulate T cell responses or EAE has not been examined thus far.

In this study, we hypothesized that exogenous administration of NLRX1 via dNP2 could alleviate disease severity of EAE by CNS penetration and hence control T cell functions. To address this, we generated and purified recombinant NLRX1 proteins including leucine-rich repeat domain (LRR) or nucleotide-binding domain (NBD) of NLRX1, conjugated with dNP2. We found that LRR, but not NBD, was required to alleviate the disease by reducing the infiltration of effector T cells into the CNS. In addition, we revealed that treatment with dNP2-LRR after adaptive immune activation could regulate CNS inflammation and EAE disease severity. This therapeutic effect is presumably mediated by inhibition of T cell activation or Th1 cell differentiation. These results suggest that the LRR domain of NLRX1 directly regulates T cell functions and therefore, it could be developed as a novel therapeutic agent for MS.

## Materials and Methods

### Purification of NLRX1 proteins

Expression plasmids in pET28a vector were constructed with dNP2 (KIKKVKKKGRKGSKIKKVKKKGRK)-LRR, dNP2-NBD, TAT (YGRKKRRQRRR)-LRR or LRR alone as inserts. The proteins were expressed in BL-21 Rosetta at 20°C overnight with 0.2 mM IPTG. Then, the cells were harvested and denatured in lysis buffer (8 M urea, 100 mM NaH_2_PO_4_, 10 mM Tris, pH 8.0). Suspended cells in the buffer were sonicated for 2 min. After centrifugation, the supernatant was collected and filtered with a 0.45 µm filter (Sartorius, Göttingen, Germany). The filtered lysate was incubated with Ni-NTA agarose (Qiagen, Hilden, Germany) beads for 30 min, and the bound proteins were then washed with a denaturing wash buffer (8 M urea, 100 mM NaH_2_PO_4_, 10 mM Tris, 80 mM imidazole, pH 8.0) and finally eluted with elution buffer (8 M urea, 100 mM NaH_2_PO_4_, 10 mM Tris, 250 mM imidazole, pH 8.0). The proteins were then desalted using a PD-10 Sephadex G-25 column (GE Healthcare, Chicago, IL, USA). To eliminate bacterial endotoxin contamination in the purified protein, the desalted protein was further incubated with 1% Triton X-114 for 30 min at 4°C. The aggregate was separated by centrifugation and this process was repeated 4 times. The proteins were finally desalted using a PD-10 Sephadex G-25 column and stored at -80°C in HBSS with 10% glycerol after quantification by Bradford assay (Bio-Rad, Hercules, CA, USA).

### Mice

All mice (C57BL/6J) were maintained in a specific pathogen-free facility at Hanyang University. The animal experiment protocol used in this study was approved by the Animal Experimentation Ethics Committee of Hanyang University. Experiments were performed according to the guidelines of the Institutional Animal Care and Use Committee of Hanyang University.

### *In vitro* delivery efficiency

Jurkat cells (4 × 10^5^ cells per well) were seeded into a 96-well plate and incubated with the recombinant proteins at 0.5, 1 or 2 µM concentrations for 1 h (dose-dependent) or 2 µM concentration for 10, 30 min, 1, 2, 6 or 12 h (time-dependent). Mouse splenocytes (1 × 10^6^ cells per well) were seeded into 24-well plates and incubated with the recombinant proteins at the indicated concentrations and at indicated time-points. After incubation, the cells were harvested and washed once with PBS. To remove membrane-bound recombinant proteins from the cells, they were trypsinized for 5 min at 37°C. After washing with PBS, the cells were fixed and permeabilized by BD fix/perm kit and intracellular proteins were stained with α-6His rabbit monoclonal antibody (Abcam, Cambridge, UK) and α-rabbit IgG Alexa Fluor 647 antibody (Invitrogen, Carlsbad, CA, USA). Intracellular fluorescence was analyzed by flow cytometry.

For western blotting, Jurkat cells were lysed with RIPA buffer (Cell Signaling Technology, Danvers, MA, USA) containing 1 mM PMSF and NaF on ice for 30 min. Total protein concentration was determined by Pierce BCA protein assay kit (Thermo Fisher Scientific, Waltham, MA, USA). After SDS-PAGE, proteins were transferred onto PVDF membranes (Bio-Rad, Hercules, CA, USA). Membranes were blocked with 5% skim milk in Tris-buffered saline containing 0.1% Tween-20. After blocking, the membranes were incubated with α-6His rabbit monoclonal antibody (Abcam) and α-rabbit IgG-HRP (Cell Signaling Technology, Danvers, MA, USA). After washing, the membranes were developed using EZ-Western Lumi Pico or Femto reagent (DoGen, Seoul, Republic of Korea). Band intensity was measured by Fusion-Solo (Vilber, Collégien, France).

### Confocal microscopy

For analyzing localization of dNP2-LRR protein in HeLa cells, 1 × 10^5^ cells per well were incubated with 0.2 µM of the recombinant protein at 37°C for 1 h. The cells were then washed three times with PBS and mitochondria were stained with 400 nM of Mitotracker cmsROX (Thermo Fisher Scientific, Waltham, MA, USA) at 37°C for 15 min. The cells were washed three times with PBS and fixed with 4% paraformaldehyde. Then, the cells were permeabilized by 0.25% Triton X-100 and intracellular proteins were stained with α-6His rabbit monoclonal antibody (Abcam, Cambridge, UK) and α-rabbit IgG Alexa Fluor 488 antibody (Invitrogen, Carlsbad, CA, USA). The fluorescence in the cytoplasm and the nucleus was analyzed using a C2si confocal microscope (Nikon, Tokyo, Japan).

### EAE

For prevention scheme and therapeutic scheme models, 10-week-old female C57BL/6 mice were purchased from Orient Bio. EAE was induced by immunization with MOG_35-55_ antigen (Hooke Labs, Lawrence, MA, USA) and 100 ng injection of pertussis toxin (PT) according to the manufacturer's instructions. In the prevention scheme model, after immunization, 50 µg of recombinant proteins were intraperitoneally injected daily, from day 2 to until they were sacrificed. In the therapeutic scheme model, 100 µg of recombinant proteins were intraperitoneally injected daily, from day 16 until they were sacrificed. In case of the semi-therapeutic model, EAE was induced by subcutaneous injection of 100 µg of MOG_35-55_ antigen (GenScript, Nanjing, China) in complete Freund's adjuvant emulsion (Chondrex, Redmond, WA, USA). At day 0 and day 2 after immunization, 200 ng of pertussis toxin (List Biological Laboratories Inc., Campbell, CA, USA) was injected intraperitoneally. Recombinant proteins (50 µg) were injected intraperitoneally on alternate days, from day 7 until day 13. Animals were scored daily for signs of clinical disease as previously described 22. Spinal cord tissues were harvested and analyzed by histology, flow cytometry and real-time polymerase chain reaction (RT-PCR).

### Histology

The spinal cord tissues were embedded in paraffin blocks and subsequently fixed with 4% paraformaldehyde. The paraffin blocks were sliced and stained with Luxol fast blue (LFB) and hematoxylin. The stained tissues were analyzed using a DMi8 microscope (Leica, Wetzlar, Germany).

### RT-PCR

Spinal cord tissues were disrupted by a homogenizer with RNAiso plus (Takara, Kusatsu, Japan) and total RNA was extracted. cDNA was synthesized with ReverTra Ace qPCR RT master mix (Toyobo, Osaka, Japan). RT-PCR was performed on a CFX Connect RT-PCR detection system (Bio-Rad, Hercules, CA, USA) using iQ SYBR Green Supermix (Bio-Rad, Hercules, CA, USA). Primers used are listed as follows: *mIl6*, F: 5'-AGGATACCACTCCCAACAGACCT-3' R: 3'-CAAGTGCATCATCGTTGTTACTAC-5'; *mTnfa,* F: 5'-CATCTTCTCAAAATTCGAGTGACAA-3' R: 3'-CCCAACATGGAACAGATGAGGGT-5'; *mIl1b,* F: 5'-GAAATGCCACCTTTTGACAGTG-3' R: 3'-TGGATGCTCTCATCAGGACAG-5'; *mIfng,* F: 5'-ATGAACGCTACACACTGCATC-3' R: 3'-CCATCCTTTTGCCAGTTCCTC-5'; *mIl17a,* F: 5'- TTTAACTCCCTTGGCGCAAAA-3' R: 3'-CTTTCCCTCCGCATTGACAC-5'; *mGmcsf,* F: 5'-GGCCTTGGAAGCATGTAGAGG-3' R: 3'-GGAGAACTCGTTAGAGACGACTT-5'; and *mActb,* F: 5'-TGTCCCTGTATGCCTCTGGT-3' R: 3'-CACGCACGATTTCCCTCTC-5'.

### Flow cytometry

The lymphocytes from the spinal cord tissues were isolated by Percoll (GE Healthcare, Chicago, IL, USA) density-gradient centrifugation and analyzed by flow cytometry. Cells were stained with fluorochrome-conjugated monoclonal antibodies; mouse anti-CD45-Pacific blue (1:1000 diluted), anti-CD4-PE-Cy7 (1:1000 diluted), anti-CD8-PerCP-Cy5.5 (1:1000 diluted), anti-CD25-PE (1:1000 diluted), anti-CD69-FITC (1:1000 diluted), anti-CD44-APC-Cy7 (1:1000 diluted), anti-CXCR3-FITC (1:200 diluted), anti-CCR6-PE-Cy7 (1:200 diluted, BioLegend, San Diego, CA, USA), anti-IFNγ-FITC (1:100 diluted), anti-IL-17A-PE (1:200 diluted), and anti-FOXP3-APC (1:400 diluted, eBioscience, San Diego, CA, USA) and anti-T-bet-PE (3 µl per sample, BD, Franklin Lakes, NJ, USA). Intracellular cytokine staining was performed using an Intracellular Fixation and Permeabilization kit according to the manufacturer's instructions (eBioscience, San Diego, CA, USA). The samples were run on a BD Canto II cytometer (BD Biosciences, San Jose, CA, USA) and the results were analyzed by FlowJo software version 10.1 (BD, Franklin Lakes, NJ, USA).

### *In vitro* T cell activation

Naïve CD4^+^ T cells were isolated using a mouse naïve CD4^+^ T Cell Isolation Kit (Miltenyi Biotec, Bergisch Gladbach, Germany) according to the manufacturer's protocol. Purified naïve CD4^+^ T cells were activated with a 1:5 ratio of anti-CD3/CD28 Dynabeads (Gibco, Co Dublin, Ireland) and incubated with the recombinant proteins at 37°C for 1 or 2 days. After incubation, the supernatant was analyzed by ELISA and the cells were stained with fluorochrome-conjugated monoclonal antibodies; mouse anti-CD4, CD62L, CD25, CD69 and CD44 (BioLegend, San Diego, CA, USA). The samples were run on a BD Canto II cytometer (BD Biosciences, San Jose, CA, USA) and the results were analyzed using FlowJo software version 10.1 (BD, Franklin Lakes, NJ, USA).

### *In vitro* T cell differentiation

Naïve CD4^+^ T cells were isolated using the mouse naïve CD4^+^ T Cell Isolation Kit (Miltenyi Biotec, Bergisch Gladbach, Germany). Purified naïve CD4^+^ T cells were cultured with 2 µg/ml plate-bound anti-CD3 (BD Biosciences, San Jose, CA, USA) and anti-CD28 (BD Biosciences, San Jose, CA, USA) antibodies in the presence of Th1, Th17, or Treg-skewing medium along with the recombinant proteins. Th1 condition: IL-2 (50 U/ml, Peprotech, Rocky Hill, NJ, USA), IL-12 (2 ng/ml, Peprotech, Rocky Hill, NJ, USA) and anti-IL-4 antibody (5 µg/ml, BD Biosciences, San Jose, CA, USA) for 4 days. Th17 condition: IL-6 (30 ng/ml, BD Biosciences, San Jose, CA, USA), TGFβ (0.5 ng/ml, R&D Systems, Minneapolis, MN, USA), IL-23 (20 ng/ml, BD Biosciences, San Jose, CA, USA), anti-IFNγ (5 µg/ml, BD Biosciences, San Jose, CA, USA) and anti-IL-4 (5 µg/ml, BD Biosciences, San Jose, CA, USA) for 4 days. Treg condition: IL-2 (100 U/ml, Peprotech, Rocky Hill, NJ, USA), TGFβ (5 ng/ml, R&D Systems, Minneapolis, MN, USA) for 3 days. After incubation, the cells were stained with fluorochrome-conjugated monoclonal antibodies; mouse anti-CD4, IFNγ, IL-17A, and FoxP3 (eBioscience, San Diego, CA, USA). Intracellular cytokine staining was performed using an Intracellular Fixation and Permeabilization kit according to the manufacturer's instructions (eBioscience, San Diego, CA, USA). The samples were run on a BD Canto II cytometer (BD Biosciences, San Jose, CA, USA) and the results were analyzed using FlowJo software version 10.1 (BD, Franklin Lakes, NJ, USA).

### Localization of dNP2-LRR in spinal cord tissues

dNP2-LRR protein (5 mg) was administered to a 7-week-old C57BL/6 female mouse intraperitoneally. After 2 h, the mouse was sacrificed and circulating blood cells were washed with 10 ml of PBS. The spinal cord tissue was harvested and was fixed in 4% paraformaldehyde overnight and dehydrated in 30% sucrose in PBS. The fixed tissue was frozen with optimal cutting temperature (OCT) compound in liquid nitrogen. The frozen block was sliced into 20 µm thin sections by cryostat and the sliced tissue on the slide was permeabilized using 0.25% Triton X-114. After blocking with 1% BSA, the tissues were stained with α-6His rabbit monoclonal antibody (Abcam, Cambridge, UK) and α-rabbit IgG Cy3 antibody (Jackson, West grove, PA, USA). The fluorescence in the spinal cord tissue was analyzed using a C2si confocal microscope (Nikon, Tokyo, Japan).

### Statistics

The data were statistically analyzed using Prism 7 software (GraphPad, San Diego, CA, USA). Tests for statistical significance were performed using two-tailed Student's *t*-test, one-way ANOVA or two-way ANOVA. Results with *P*-values less than 0.05 were considered as statistically significant.

## Results

### Generation of NLRX1 recombinant proteins conjugated with dNP2

**Figure 1:**
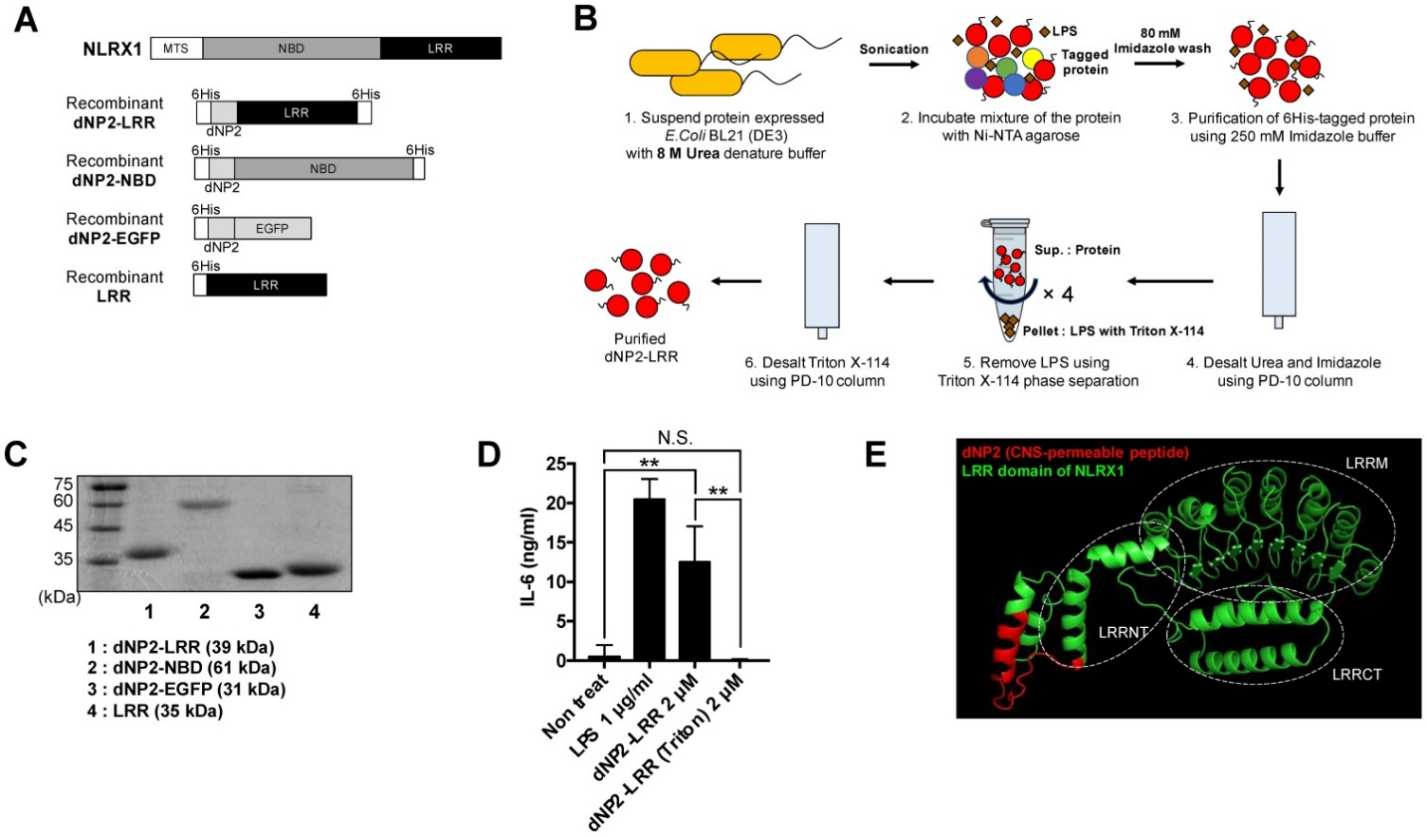
** Generation of dNP2-conjugated NLRX1 proteins.** (A) The structure dNP2-LRR, dNP2-NBD, dNP2-EGFP and LRR DNA construct. (B) Purification procedure of dNP2-LRR protein for high purity. (C) Purified proteins were analyzed by 12% SDS-PAGE gel. (D) Endotoxin contamination assay with RAW264.7 cells. The cells were incubated with LPS, dNP2-LRR or dNP2-LRR (Triton phase separation) for 12 h. IL-6 production in the culture supernatant was measured by ELISA. (E) 3D structure prediction of the dNP2-LRR. *n* = 2 per group and *error bars* indicate S.D. **P<0.01. *N.S.*, not significant.

In previous studies, we have reported various, efficient human-derived cell-penetrating peptides such as LPIN 23, 2pIL-1αNLS 24, Iduna 25, dNP2 12, and AP 26. Particularly, dNP2 is a BBB-permeable peptide, which enables delivery of T cell regulatory proteins into the CNS to inhibit spinal cord inflammation in EAE. NLRX1 is composed of an LRR domain and an NBD with an N-terminal mitochondrial targeting sequence. Therefore, we generated DNA constructs that could express the NLRX1 functional motifs (LRR, NBD) conjugated with dNP2, as recombinant proteins (Figure 1A). We expressed the proteins in *E.coli* and the 6His-tagged proteins were purified by affinity chromatography under denaturing conditions and the bacterial LPS was removed by Triton X-114 phase separation method (performed 4 times) 27, 28 (Figure 1B), and then analyzed by SDS-PAGE (Figure 1C). LPS-removed dNP2-LRR treatment of RAW264.7 macrophages for 12 h did not produce IL-6 cytokine, However, treatment of macrophages with an equivalent amount of protein without endotoxin removal process showed a significant increase in IL-6, which implies that endotoxin contamination stimulates macrophages (Figure 1D). Recombinant dNP2-LRR protein structure was predicted using SparksX (available online: http://sparks-lab.org/yueyang/server/SPARKS-X/) (Figure 1E). The alpha helical structure of dNP2 has been highlighted in red with the continuing LRR domain of NLRX1 in green, which has been overlaid to match with a previously reported LRR structure 29 suggesting that conjugation of dNP2 might not significantly affect structural changes in the LRR domain.

### dNP2 enables the delivery of LRR domain of NLRX1 into T cells

**Figure 2:**
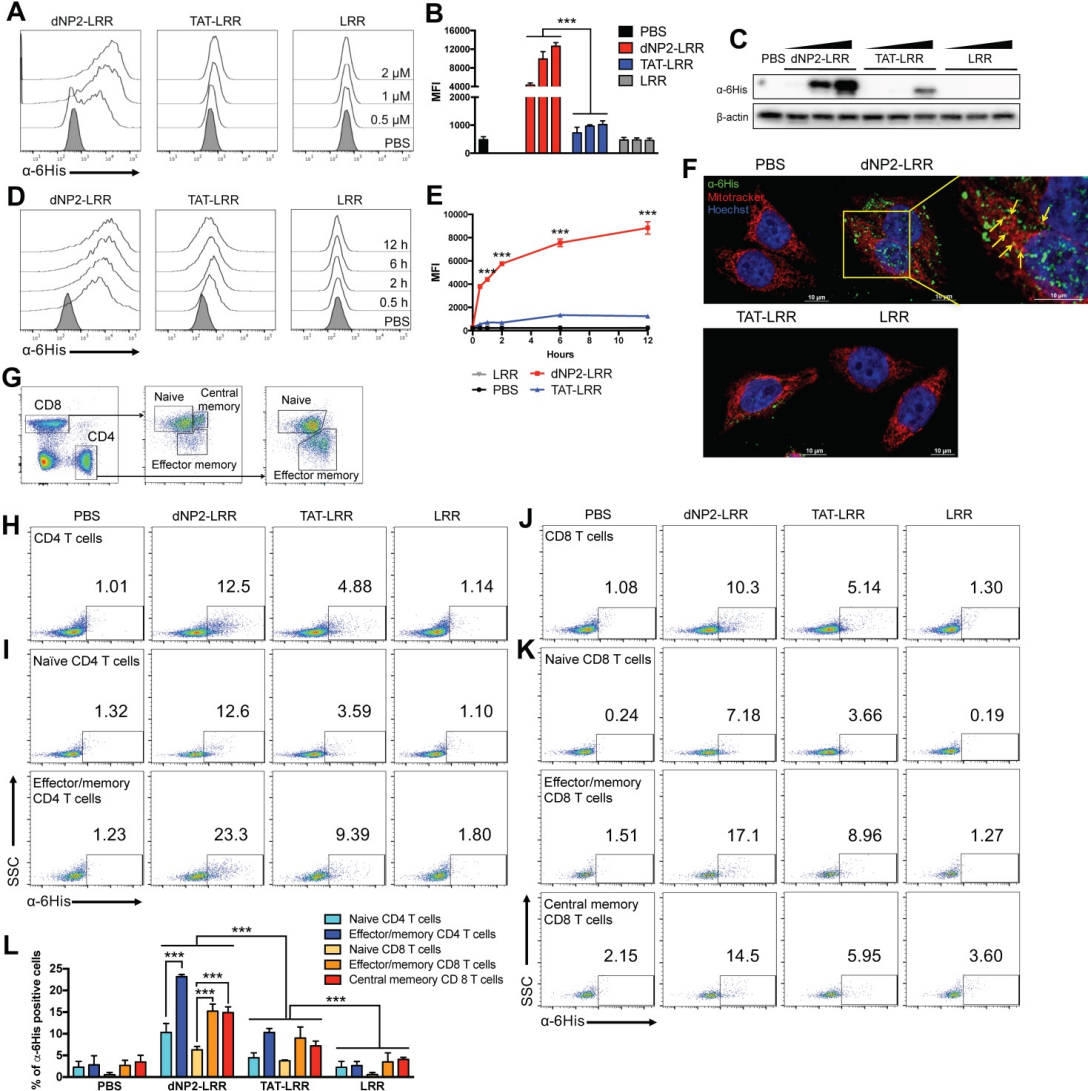
** Intracellular delivery efficiency of dNP2-LRR.** (A-B) Intracellular delivery efficiency in Jurkat T cells was analyzed after incubation with 0.5, 1, 2 μM of recombinant proteins for 1 h. Intracellular fluorescence was analyzed by flow cytometry. (C) Jurkat T cells were lysed and the intracellular LRR protein was analyzed by western blot. (D-E) Jurkat T cells were incubated with 2 μM of each recombinant protein for 0-12 h. Intracellular fluorescence was analyzed by flow cytometry. (F) Intracellular localization of dNP2-LRR in HeLa cells. The fluorescence was visualized by confocal microscopy. (G) The gating strategy of T cells in splenocytes. Total splenocytes were incubated with 2 μM of each recombinant proteins for 1 h. Intracellular delivery efficiency was analyzed by flow cytometry after stained with specific markers and intracellular proteins. Subsets of (H-I) CD4 T cells, (J-K) CD8 T cells were differentially analyzed. (L) Proportion of the cells with intracellular LRR protein was analyzed by 3 independent experiments. *n* = 3 per group and *error bars* indicate S.D. ***P<0.001. *MFI*, mean fluorescence intensity

To confirm the cell permeability of the purified proteins in T cells, 0.5-2 µM of dNP2-LRR, TAT-LRR and LRR proteins were incubated with Jurkat T cells, which is a human T lymphocyte cell line, and the intracellular protein level was determined by flow cytometry using anti-6His primary antibody and Alexa Fluor 647-labeled anti-rabbit secondary antibody (Figure 2A-B). dNP2 conjugation to LRR domain of NLRX1 showed dose-dependent increase in efficiency of intracellular delivery, which was significantly higher than either TAT-LRR or LRR alone. This was also confirmed by western blot analysis (Figure 2C). The delivery efficiency of dNP2-LRR was significant at 0.5 h of treatment and gradually increased up to 12 h of incubation, while TAT-LRR showed significant delivery efficiency at 2 h with slight increase up to 6 h, and subsequently decreased by 12 h of treatment. This suggests the potent intracellular protein transduction ability of dNP2-LRR compared to TAT in T cells (Figure 2D-E). To visualize the cellular localization of the recombinant proteins, we incubated the proteins with HeLa cells. In HeLa cells, dNP2-LRR (0.2 µM) was localized in both cytoplasm and nucleus of the cells and a small portion of internalized proteins were co-localized with the mitochondria. However, only few puncta were detected in the cytosol of TAT-LRR-treated cells with comparable background of LRR and PBS (Figure 2F) suggesting that dNP2 enables efficient localization of the LRR domain of NLRX1 in various regions. Due to the difficulty of drug delivery in primary T cells, we examined its transduction efficiency in mouse splenocytes at a concentration of 2 µM for 1 h. We classified T cell population as CD62L^high^CD44^low^ naïve CD4^+^ T cells, CD62L^high^CD44^low^ effector/memory CD4^+^ T cells and CD8^+^T cell subsets as CD62L^high^CD44^low^ naïve CD8^+^T cells, CD62L^low^CD44^high^ effector/memory CD8^+^ T cells and CD62L^high^CD44^high^ central memory CD8^+^ T cells (Figure 2G). In total CD4^+^ T cells, dNP2 showed increased proportion of cells with intracellular LRR protein than other controls such as TAT-LRR (Figure 2H). Its delivery efficiency in effector/memory cells was significantly higher than in naïve cells (Figure 2I) as reported previously 30. Similar to CD4^+^ T cells, dNP2-LRR was efficiently delivered into CD8^+^ T cells with higher delivery efficiency than TAT-LRR (Figure 2J) and there was comparably higher amount of protein in both effector/memory and central memory cells than in naïve cells (Figure 2K-L). These results suggest that we successfully generated highly efficient T cell-deliverable NLRX1 recombinant proteins for functional evaluation.

### dNP2-LRR, but not NBD, ameliorates EAE disease progression

**Figure 3:**
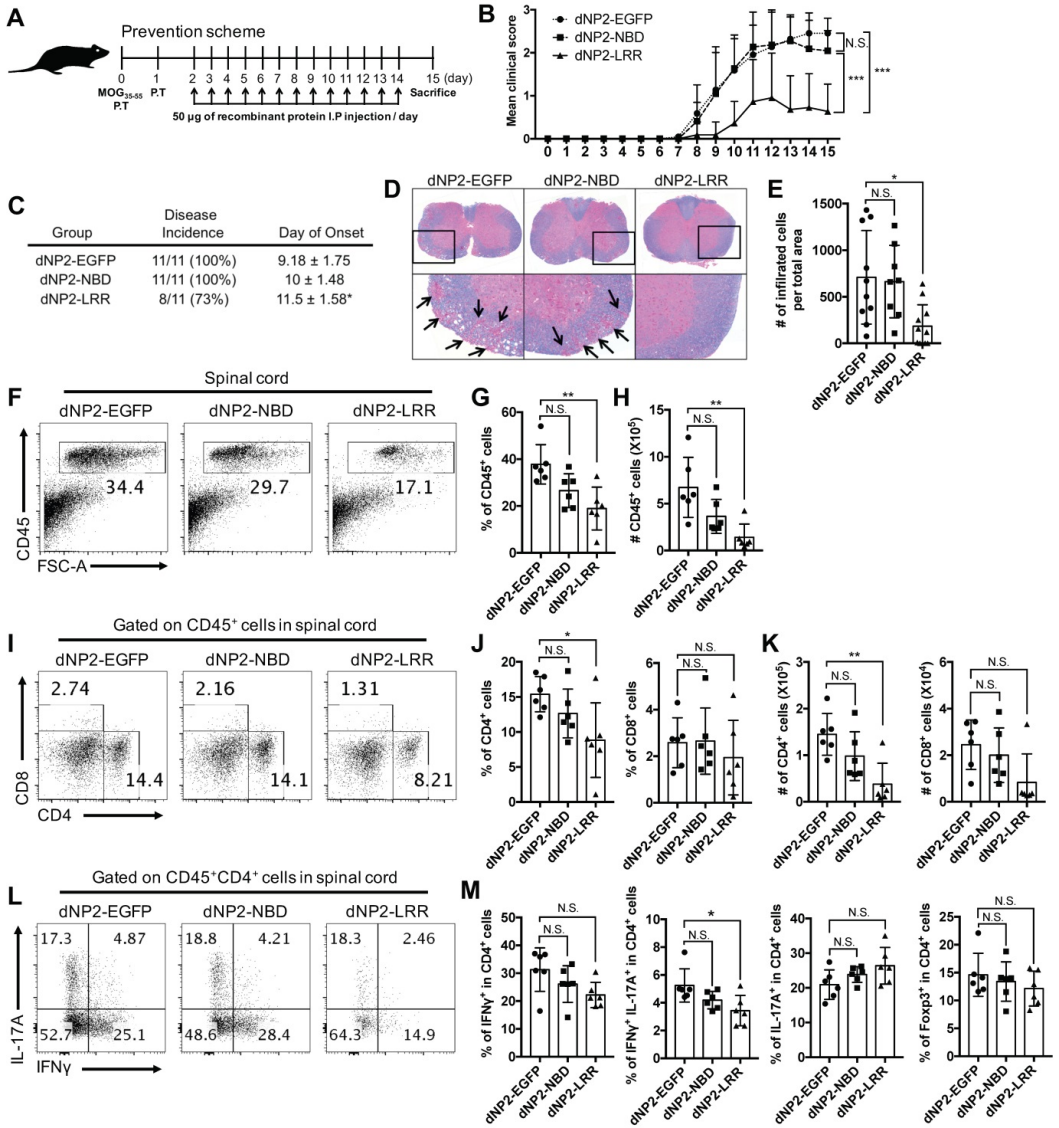
** dNP2-LRR ameliorates CNS inflammation and disease severity of EAE but not dNP2-NBD or dNP2-EGFP.** (A) Prevention scheme of EAE treatment. (B) Clinical score and (C) disease incidence of prevention scheme treatment was analyzed until day-15. (D) Spinal cord tissues were stained with LFB and hematoxylin to analyze inflammation and demyelination. (E) The number of infiltrated cells in the spinal cord tissue in the sectioned slide was counted under microscope (F-G) The frequency or (H) absolute number of CD45^+^ cells in percoll-isolated total cells from the spinal cord tissues was analyzed by flow cytometry. (I-J) The frequency or (K) absolute number of CD4^+^ or CD8^+^ cells were analyzed. (L-M) The frequency of IFNγ^+^, IFNγ^+^IL-17A^+^, IL-17A^+^, or Foxp3^+^ CD4 T cells was analyzed. *n* = 11 per group and *error bars* indicate S.D. *P <0.05, **P<0.01 and ***P<0.001. *N.S.*, not significant.

Since NLRX1 deficient mice had more severe experimental autoimmune encephalomyelitis (EAE) than wild-type mice 31, and dNP2 showed remarkable protein delivery efficiency in T cells, we hypothesized that exogenous administration of NLRX1 proteins might be able to control EAE disease progression. dNP2-LRR (50 µg) and dNP2-NBD proteins were intraperitoneally administered daily from day 2 in MOG_35-55_ immunization model and clinical symptoms of the mice were monitored until the day of sacrifice, i.e. day 15 (Figure 3A). The clinical score (Figure 3B) and the incidence (Figure 3C) was significantly reduced by dNP2-LRR treatment while NBD or EGFP did not show disease amelioration. Histological analysis indicated significant reduction of demyelination (Figure 3D) and decreased number of infiltrated cells in the spinal cord (Figure 3E) suggesting that dNP2-LRR, but not NBD, of NLRX1 has a regulatory function in EAE progression. Furthermore, analysis of the isolated spinal cord cells demonstrated that the number of CD45^+^ immune cells were significantly reduced in dNP2-LRR treated mice (Figure 3F-H). Within the population, the proportion and the number of CD4^+^ T cells were also significantly decreased by dNP2-LRR treatment (Figure 3I-K). In CD4^+^ T cells, the proportion of IFNγ-producing cells were significantly decreased but not IL-17A-producing cells (Figure 3L-M) implying a possible *in vivo* mechanism of inhibition of IFNγ production by dNP2-LRR. These results suggest that administration of recombinant LRR domain, but not NBD, of NLRX1 via dNP2 was sufficient to control EAE.

### dNP2-LRR ameliorates EAE disease progression after the onset of adaptive immune response

**Figure 4:**
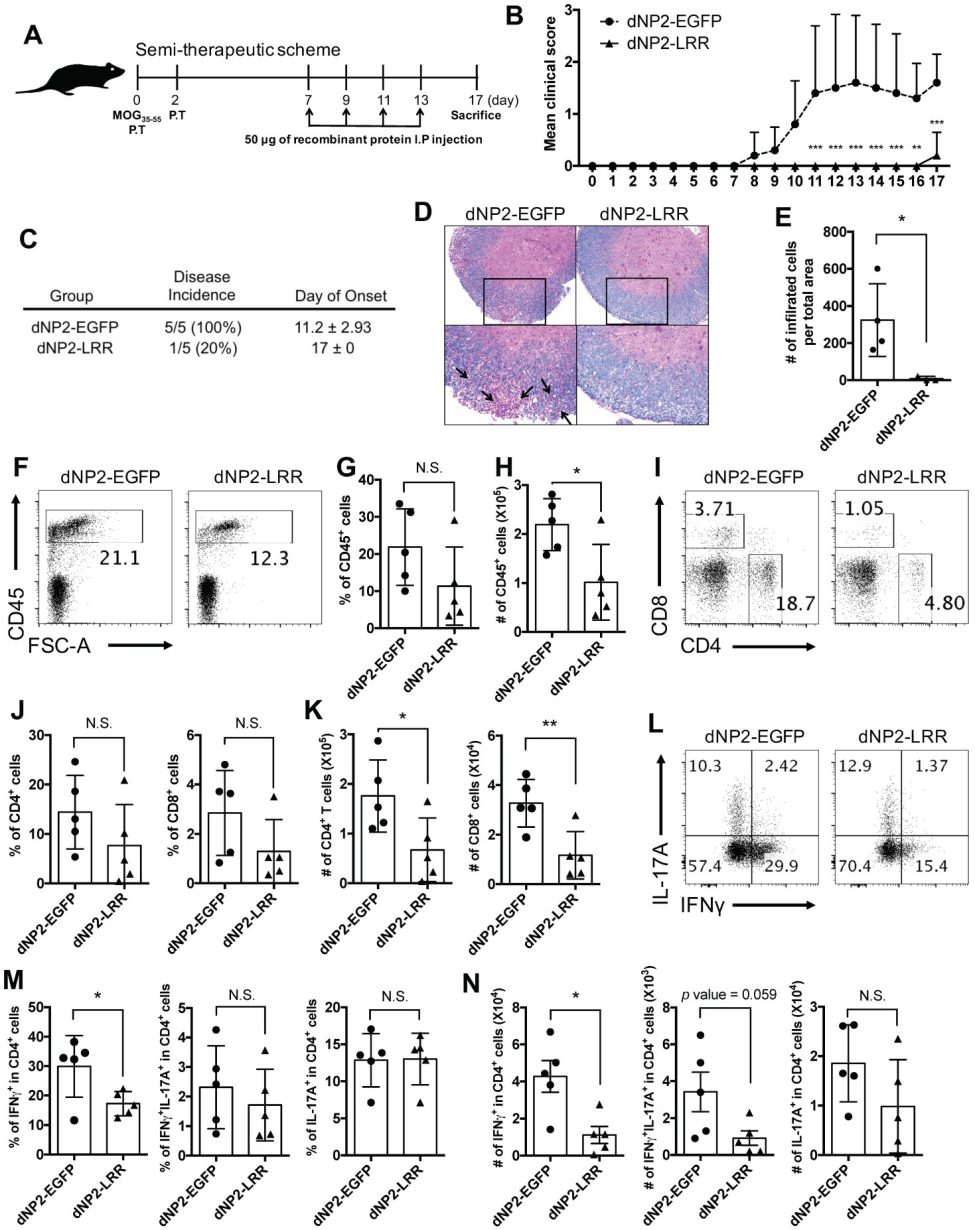
** dNP2-LRR inhibits EAE disease progression after onset of adaptive immune response.** (A) Semi-therapeutic scheme of EAE treatment. (B) Clinical score and (C) disease incidence of semi-therapeutic treatment was analyzed until day-17. (D) Spinal cord tissues were stained with LFB and hematoxylin to analyze inflammation and demyelination. (E) The number of infiltrated cells in the spinal cord tissue in the sectioned slide was counted under microscope. (F-G) The frequency or (H) absolute number of CD45^+^ cells in Percoll-isolated total cells from the spinal cord tissues was analyzed by flow cytometry. (I-J) The frequency or (K) absolute number of CD4^+^ or CD8^+^ cells were analyzed. (L-M) The frequency and (N) the number of IFNγ or IL-17A producing cells were analyzed. *n* = 5 per group and *error bars* indicate S.D. *P <0.05, **P<0.01 and ***P<0.001. *N.S.*, not significant.

Based on successful prevention of EAE by dNP2-LRR treatment, we further examined its therapeutic effects on immunized mice. The proteins were administered to MOG-immunized mice after the onset of adaptive immune response (day 7) on alternate days until day 13, and the mice were analyzed on day 17 (Figure 4A). The dNP2-EGFP-treated group of control mice showed the onset of disease on day 8 which rapidly progressed by day 11 sustaining an average score of 1.5 until day 17, while the dNP2-LRR-treated group showed no signs of disease until day 16 (Figure 4B). By the end of the experiment on day 17, only 1 out of 5 mice of the dNP2-LRR-treated group showed reduced tail strength as observed in EAE disease incidences (Figure 4C) suggesting significant inhibition of EAE by dNP2-LRR treatment. Spinal cord tissue histology showed that neuronal damage (Figure 4D) and cellular infiltration (Figure 4E) were significantly reduced in the dNP2-LRR-treated group compared to the control group. Due to the importance of IFNγ production by T cells, and as both Th1 and Th17 cells contribute to EAE 32, 33, we analyzed spinal cord infiltrating cells. The proportion and the absolute number of CD45^+^ cells in the isolated spinal cord cells from the mice were significantly decreased by dNP2-LRR treatment compared to dNP2-EGFP treatment (Figure 4F-H). The proportion of both CD4^+^ and CD8^+^ T cells (Figure 4I-J) showed reduced pattern upon dNP2-LRR treatment with increased number of infiltrating CD4^+^ and CD8^+^ T cells (Figure 4K). Importantly, the proportion and number of IFNγ-producing CD4^+^ T cells in spinal cord-infiltrating CD4 T cells was significantly reduced (Figure 4L-N). CNS-infiltrating T cell analysis collectively suggests that dNP2-LRR ameliorates EAE disease progression even after adaptive immune activation, presumably due to reduced IFNγ-producing ability or infiltration of Th1 cells, in the spinal cord.

### dNP2-LRR inhibits T cell activation and regulates Th1 differentiation

**Figure 5:**
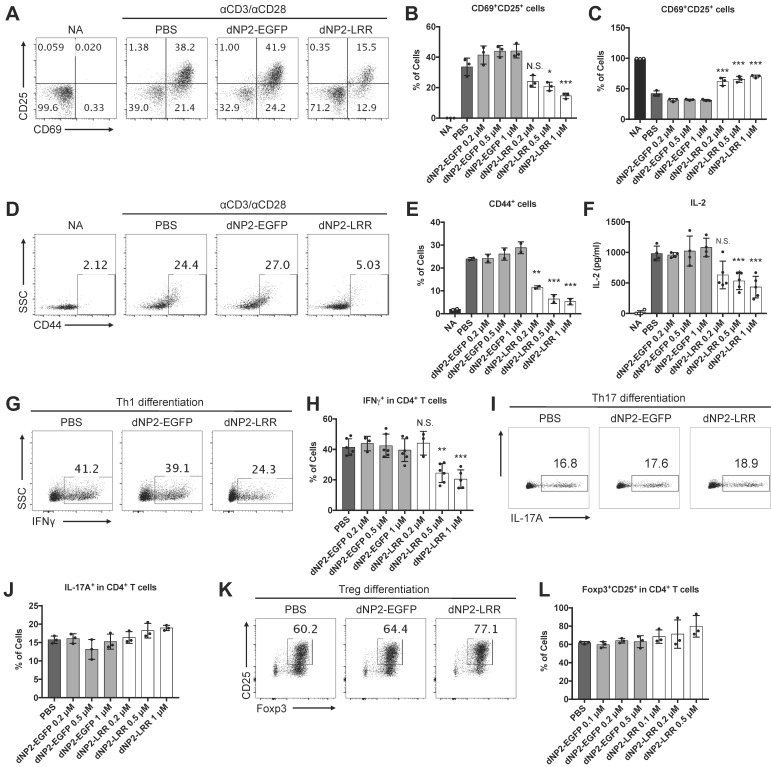
** dNP2-LRR inhibits T cell activation and specifically inhibits Th1 differentiation.** (A) The surface expression level of CD25 and CD69 in activated CD4 T cells was analyzed after incubation with 1 μM of dNP2-LRR or dNP2-EGFP or PBS. The frequency of (B) CD69^+^CD25^+^ activated cells or (C) CD69^-^CD25^-^ non-activated cells at indicated concentration of dNP2-LRR. (D-E) The level of CD44 in activated CD4 T cells. (F) IL-2 production in the culture supernatant was measured by ELISA. (G-H) The frequency of IFNγ producing cells in Th1 differentiation with dNP2-LRR, dNP2-EGFP or PBS was analyzed by flow cytometry. (I-J) The frequency of IL-17A producing cells in Th17 differentiation. (K-L) The frequency of Foxp3^+^CD25^+^ regulatory T cells in iTreg differentiation. MACS-sorted naïve CD4 T cells were used in all the experiments. *n* = 3 to 6 per group and *error bars* indicate S.D. *P <0.05, **P<0.01 and ***P<0.001. *NA*, non-activated; *N.S.*, not significant.

Given the significant inhibition of EAE disease with reduced infiltration of T cells, especially IFNγ-producing CD4 T cells, in the spinal cord, we hypothesized that dNP2-LRR might directly regulate T cell function. To address this, we utilized MACS (magnetic activated cell sorting)-purified naïve CD4 T cells (CD4^+^CD44^-^) with anti-CD3/28 antibody stimulation. The surface expression levels of T cell activation markers including CD69 and CD25 were significantly reduced, and the frequencies of inactivated (CD69^-^CD25^-^) populations were significantly increased in the dNP2-LRR-treated T cells compared to either dNP2-EGFP- or PBS-treated groups (Figure 5A-C). In addition, the expression of another marker of CD44 was reduced in activated T cells by dNP2-LRR (Figure 5D-E) suggesting that dNP2-LRR treatment of T cells inhibited their activation. IL-2 production in the culture supernatant was also significantly reduced in a dose-dependent manner by dNP2-LRR treatment (Figure 5F) suggesting that intracellular transduction of LRR domain of NLRX1 could directly regulate T cell activation and cytokine production. We further examined the effect of dNP2-LRR on the differentiation of effector T cells such as Th1, Th17, and Treg cells, which play an important role in EAE. Naïve CD4 T cells were treated with dNP2-LRR or control protein and differentiated into Th1, Th17, and Treg cells *in vitro*. When the naïve T cells were differentiated into Th1 cells by dNP2-LRR treatment, the population of IFNγ producing cells was significantly reduced (Figure 5G-H). However, there was no difference in Th17 differentiation (Figure 5I-J) or differentiation of regulatory T cells (Foxp3^+^CD25^+^CD4^+^ cells, Figure 5K-L). This result was not due to the toxicity of dNP2-LRR in T cells (Figure S1A-B). These findings collectively suggest that dNP2-LRR specifically regulates Th1 differentiation, which could be a possible mechanism in regulating EAE disease.

## Discussion

Multiple sclerosis is an autoimmune disease caused by inflammation, which occurs in the CNS when immune cells recognize the proteins on neurons as antigens. Although this disorder is a significant health burden, there are no therapeutic drugs available yet for its treatment 1, 34. Recently, the US-FDA approved 4 different drugs to treat MS including ocrelizumab (anti-CD20 on B cells), siponimod (S1PR modulator on T cells), cladribine (targets highly proliferating cells such as T and B cells), and diroximel fumarate (mechanism of action is unknown). While several recently developed MS drugs are targeting adaptive immune cells, which migrate to CNS tissues, the additional ability to penetrate BBB would be beneficial to regulate CNS diseases 35, 36.

Various carriers have been developed to deliver cargo into the CNS by penetrating the BBB, such as nanoparticles, liposomes, and chemical bonding 8-11. In a previous study, we identified dNP2 as a novel BBB penetrating peptide, which could deliver a cargo protein into the CNS tissue. In real-time, *in vivo* multi-photon confocal microscopy of mouse brain, dNP2-conjugated dTomato could be diffused out from blood vessels of the brain, but it was not possible by either TAT or Hph-1. Moreover, dNP2-ctCTLA-4, but not Hph-1-ctCTLA4, significantly ameliorated EAE 12. In addition, we confirmed that 11R-dTomato could not be delivered into the spinal cord and, dNP2-VIVIT significantly ameliorated EAE progression compared to 11R-VIVIT 13. In the current study, we also confirmed *in vivo* localization of dNP2-LRR in the spinal cord (Figure S2). dNP2-LRR, but not TAT-LRR or LRR alone, could inhibit EAE progression (Figure S3A-D). These results collectively suggest that dNP2, via a cargo molecule, is required for efficient modulation of EAE disease due to its high efficiency of BBB penetration.

The potent delivery efficiency of dNP2 seems to be not mediated by specific mechanisms other than reported mechanisms of CPP (cell-penetrating peptide), which are mediated by heparan sulfate proteoglycan (HSPG) and lipid-raft mediated endocytosis 12, 37, 38. The activated T cells express increased negatively charged HSPG on their surface compared to naïve T cells 39 and the cytoplasmic area in these cells is enlarged after activation. We speculate that this could be the reason why dNP2-LRR was more efficiently delivered into activated T cells. In addition, we could not observe significant differences in the migration (Figure S4A-D) or proliferation (Figure S5A-B) of draining lymph nodes implying that dNP2-LRR might mainly function in the inflammatory region. Since dNP2 does not have specificity, it cannot be excluded that dNP2-LRR might affect other immune cells including macrophages, monocytes, etc. Further study is required to elucidate the detailed mechanism of LRR domain in regulating both innate and adaptive immune cells during EAE progression.

A recent study has shown that NLRX1 may be an important negative regulator in the pathogenesis of EAE. NLRX1^-/-^ mice developed more severe EAE than WT mice and NLRX1^-/-^ microglia produced more IL-6 and CCL2 with higher expression of MHCII than WT 20. Another recent report has shown that transfer of 2D2 T cells into NLRX1^-/-^RAG^-/-^ mice induced more severe EAE than RAG^-/-^ mice 21. In addition, loss of NLRX1 exacerbated tissue damage upon brain injury via increased NF-κB signaling by CD11b^+^ cells including microglia 40. During inflammation, extracellular glutamate induced excitotoxic cell death and worsened the condition, and NLRX1 could inhibit excess glutamate release and potentiate glutamate uptake by astrocytes 41. These results collectively suggest the functional role of NLRX1 in innate immune cells contributing to EAE. Here, we first addressed the possible therapeutic potential of NLRX1 in EAE model by generating chimeric recombinant protein. In addition, we showed its regulatory role in T cells. We examined the therapeutic potential of dNP2-LRR treatment at the peak stage of EAE suggesting that it indeed shows significant therapeutic effects on EAE progression even after adaptive immune activation (Figure S6). Therefore, we suggest that NLRX1 plays a significant role in controlling T cell response as well as innate immune cells.

NLRX1 is composed of three domains: a mitochondrial targeting sequence, a nucleotide-binding domain (NBD) and a leucine-rich repeat (LRR) domain. A previous study has shown that truncation of LRR domain results in dysfunctional regulation of MAVS-mediated IRF3 signaling 14, and overexpression of LRR domain inhibited NF-κB signaling. Mechanistically, the LRR domain inhibited auto-phosphorylation of IKKβ by direct interaction with activated IKKβ 18. These results revealed that the LRR domain of NLRX1 is an important region as a negative regulator of immune responses. We also demonstrate in this study that the LRR domain, but not NBD, has a significant inhibitory function in regulating T cell functions and EAE. Recently, the fatty acids such as punicic acid (PUA) was suggested as possible ligands of the C-terminal region of NLRX1, which includes LRR domain to inhibit NF-κB activation 42 and a chemical compound which is a activator of NLRX1, NX-13 was identified showing alleviated colitis by inhibition of Th1 and Th17 differentiation 43. We also proved intracellular delivery of LRR protein could be able to inhibit Th1 differentiation, which may be potentiated with ligand co-treatment. dNP2-LRR could regulate T cell activation and Th1 differentiation presumably by regulating NF-κB signaling with slightly reduced T-bet expression (Figure S7). This also might be related to metabolic regulation such as decreased glucose uptake by LRR in Th1 cells based on the previous report suggesting NLRX1^-/-^ showed increase of glucose uptake and Th1 differentiation 19.

In this study, we demonstrate the ability of NLRX1 to control T cell functions and CNS autoimmune disease. By generating the BBB-penetrating dNP2-LRR protein, we demonstrated its therapeutic potential in EAE, a mouse model of MS. In particular, the LRR domain of NLRX1 could control T cell activation, cytokine production, and Th1 differentiation. dNP2-LRR has the clinical potential to be a novel drug molecule for treating T cell-mediated autoimmune diseases such as multiple sclerosis.

## Conclusion

In summary, we generated a recombinant LRR domain of NLRX1 protein conjugated with the BBB-penetrating peptide dNP2 (dNP2-LRR), which directly inhibits the functions of effector T cells and ameliorates disease severity of EAE with reduced T cell infiltration and IFNγ production in the CNS. We expect that dNP2-LRR could be further developed as a novel therapeutic agent for treating CNS autoimmune diseases such as multiple sclerosis.

## Supplementary Material

Supplementary figures.
