# Supplementary material for: LRR domain of NLRX1 protein delivery by dNP2 inhibits T cell functions and alleviates autoimmune encephalomyelitis
Source: Theranostics. 2020 Feb 10;10(7):3138–50. doi: 10.7150/thno.43441 (PMC7053182; doi:10.7150/thno.43441)
Supplement: Supplementary file 1 — Supplementary figures. [file thnov10p3138s1.pdf]

Supplemental Information

LRR domain of NLRX1 protein delivery by dNP2 inhibits T cell functions and alleviates autoimmune encephalomyelitis

Ja-Hyun Koo[1,2], Do-Hyun Kim[1,2], Donghun Cha[1,2], Min-Jong Kang[3] and Je-Min Choi[1,2,4*]

[1]Department of Life Science, College of Natural Sciences, Hanyang University, Seoul 04763, Republic of Korea; [2]Research Institute for Natural Sciences, Hanyang University, Seoul 04763, Republic of Korea; [3]Section of Pulmonary, Critical Care and Sleep Medicine, Department of Internal Medicine, Yale University School of Medicine, New Haven, CT 06520, USA; [4]Research Institute for Convergence of Basic Sciences, Hanyang University, Seoul 04763, Republic of Korea

*Corresponding Author
Je-Min Choi, Ph.D.
Professor of Immunology, Department of Life Science, Hanyang University
222 Wangsimni-ro, Seongdong-gu, Seoul 04763, Republic of Korea
Tel: +82-2-2220-4765 Fax: +82-2-2298-0319
Email: jeminchoi@hanyang.ac.kr

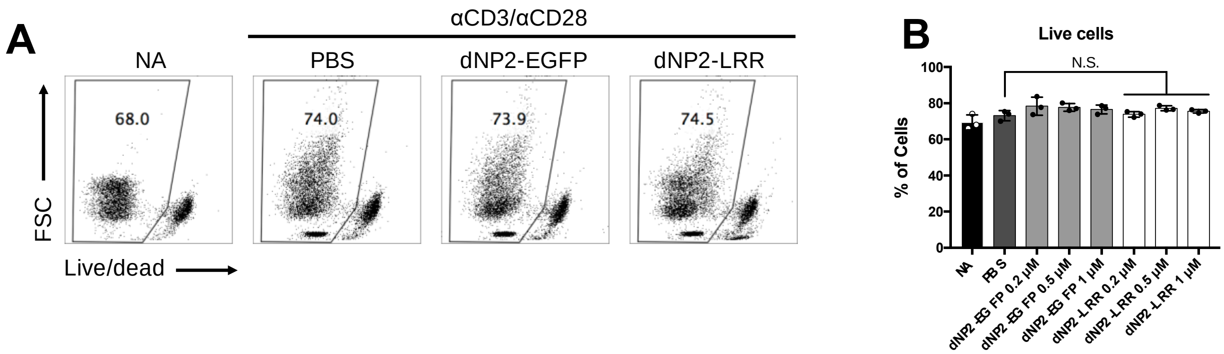

Figure S1. Cytotoxicity of dNP2-LRR.

(A) Gating of live cells using live/dead staining kit. (B) The proportion of live T cells was analyzed after incubation with dNP2-LRR or dNP2-EGFP or PBS for 24 h. n = 3 per group and error bars indicate S.D. NA, non-activated; N.S., not significant.

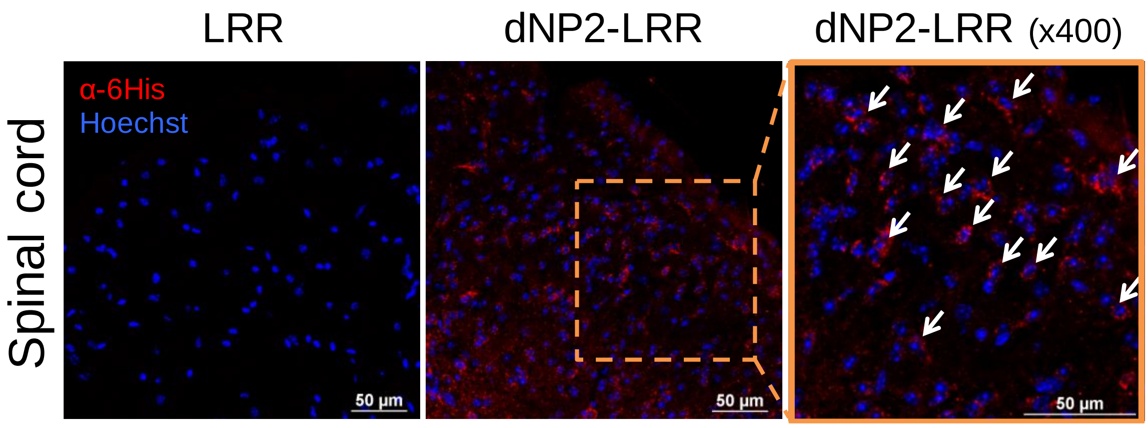

Figure S2. ***In vivo*** **localization of dNP2-LRR in the spinal cord.**

dNP2-LRR (5 mg) or LRR were injected intraperitoneally and tissues were harvested after 2 h. Intracellular proteins were visualized by confocal microscopy with 200× magnification.

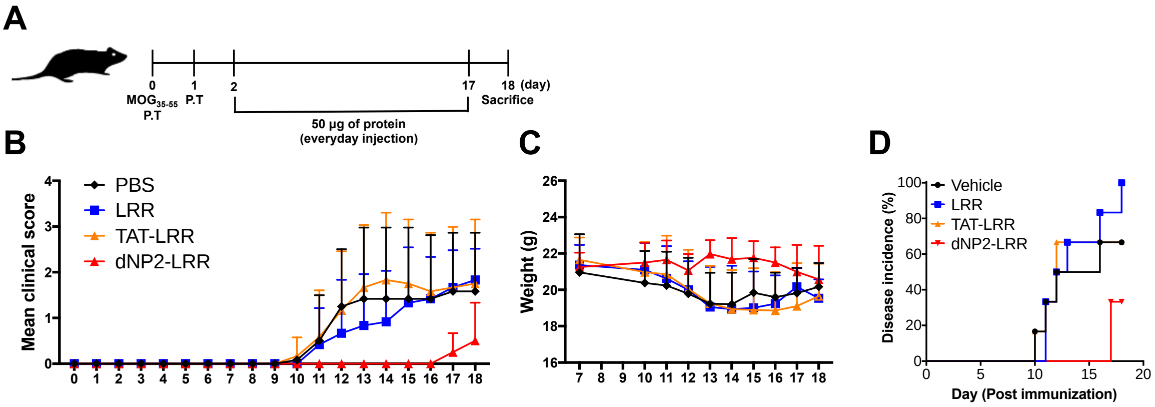

Figure S3. dNP2-LRR ameliorates EAE, but not LRR or TAT-LRR.

(A) Scheme of EAE treatment. (B) Clinical score, (C) weight and (D) disease incidence.

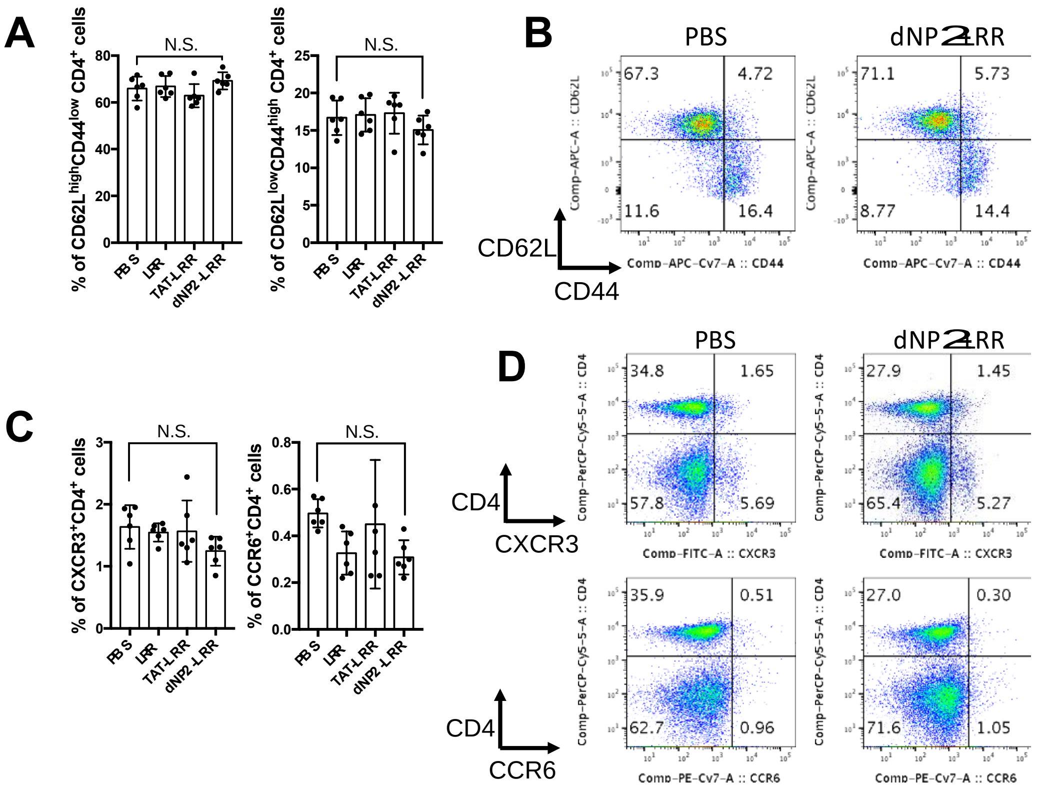

Figure S4. dNP2-LRR does not affect migration-related molecules of T cells in inguinal lymph nodes of EAE mice.

(A-B) The proportion of CD62L$_{high}$CD44$_{low}$ or CD62L$_{low}$CD44$_{high}$ CD4$_{+}$ T cells in inguinal lymph nodes of EAE mice were analyzed. (C-D) The proportion of CXCR3$_{+}$CD4$_{+}$ or CCR6$_{+}$CD4$_{+}$ cells in inguinal lymph nodes of EAE mice were analyzed by flow cytometry.

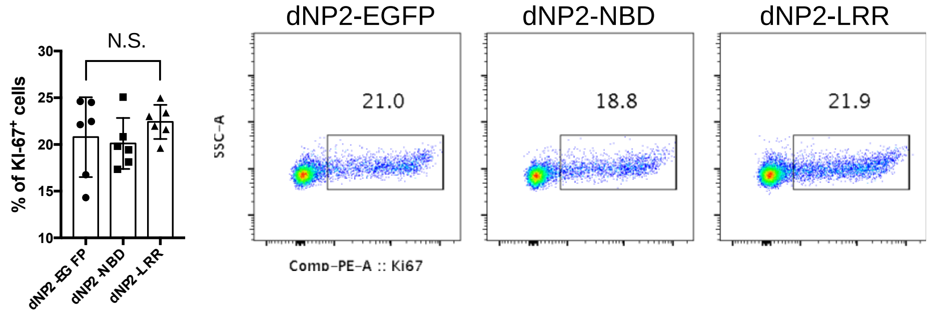

Figure S5. dNP2-LRR does not affect a proliferation-related molecule of T cells in inguinal lymph nodes of EAE mice.

(A-B) The proportion of Ki-67+ CD4+ T cells in inguinal lymph nodes of EAE mice were analyzed.

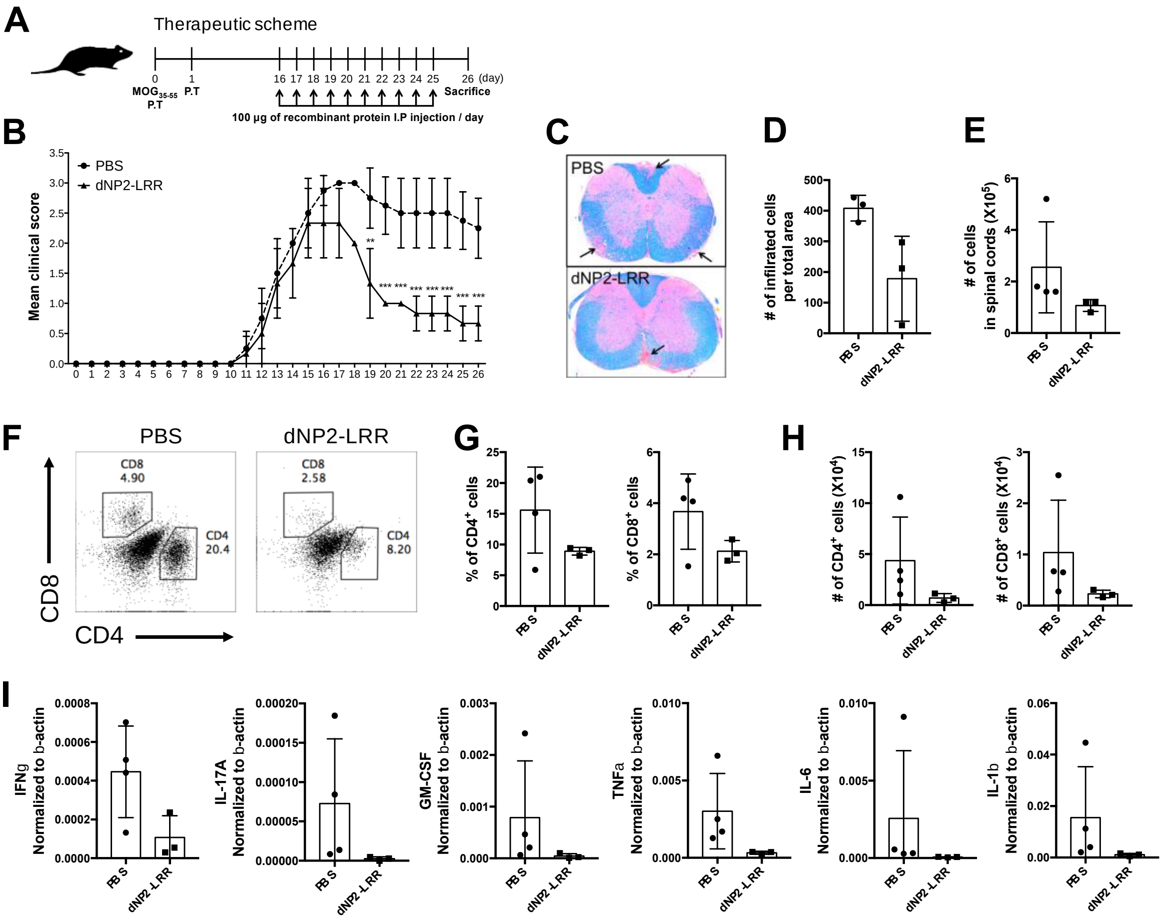

Figure S6. Therapeutic effect of dNP2-LRR treatment at the peak stage of EAE.

(A) Therapeutic scheme of EAE treatment. (B) Clinical score of therapeutic treatment was analyzed until day 26. (C) Spinal cord tissues were stained with LFB and hematoxylin to analyze demyelination and inflammation. (D-E) The number of infiltrated cells in the spinal cord tissue section was counted under the microscope. (F-G) The proportion or (H) absolute number of CD4+ or CD8+ cells were analyzed. (I) mRNA levels of inflammatory cytokines in the spinal cord were analyzed by RT-PCR. n = 3 to 4 per group and error bars indicate S.D. **P* <0.05, ***P*<0.01 and ****P*<0.001.

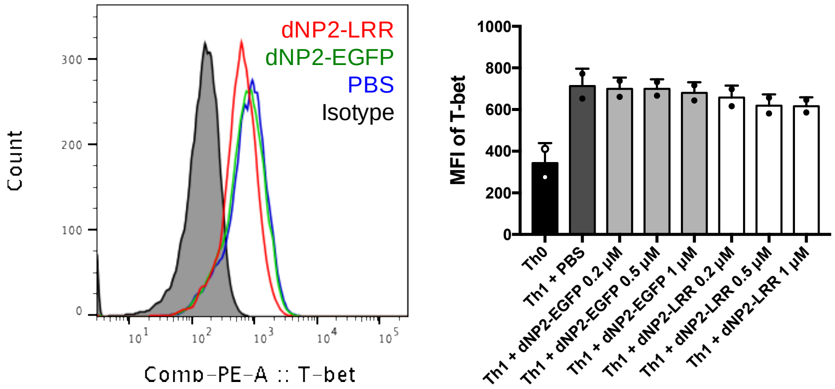

Figure S7. dNP2-LRR slightly reduced the expression of T-bet in Th1. Naïve CD4 T cells were isolated from murine spleen by MACS and co-incubated with dNP2-LRR, dNP2-EGFP or PBS in Th1 skewing medium. T-bet levels (median fluorescence intensity
